# Translation and Cross-Cultural Adaptation of SATIS-Stroke for Use in Brazil: A Satisfaction Measure of Activities and Participation in Stroke Survivors

**DOI:** 10.1155/2019/8054640

**Published:** 2019-02-18

**Authors:** Gabriela Santos Pereira, Soraia Micaela Silva, Cíntia Elord Júlio, Jean-Louis Thonnard, Edouard Bouffioulx, João Carlos Ferrari Corrêa, Fernanda Ishida Corrêa

**Affiliations:** ^1^Postgraduate Program in Rehabilitation Sciences, University Nove de Julho (UNINOVE), São Paulo, SP, Brazil; ^2^Institute of NeuroScience, Universite Catholique de Louvain, Brussels, Belgium; ^3^Département de Kinésithérapie et d'Ergothérapie, Haute École Louvain en Hainaut, Charleroi, Belgium

## Abstract

**Introduction:**

SATIS-Stroke was developed to measure satisfaction regarding activities and participation among stroke survivors based on the concepts contained in the International Classification of Functioning, Disability, and Health. However, this measure is only available in English and French.

**Objective:**

Perform the translation and cross-cultural adaptation of SATIS-Stroke to Brazilian Portuguese and test the preliminary reliability of this measure.

**Methods:**

The translation process followed standardized guidelines and consisted of six phases: initial translation, back-translation, analysis of expert committee, test of final version, submission, and assessment of all written reports. To test the preliminary test-retest reliability, the measure was administered by a single observer on two occasions with an interval of 7 to 14 days for the determination of intraobserver agreement and administered again by a second observer for the determination of interobserver agreement. Reliability was analyzed using the intraclass correlation coefficient (ICC_2,1_) and respective 95% confidence intervals (CI).

**Results:**

All stages of the cross-cultural adaptation process were respected and the final translated version of SATIS-Stroke exhibited semantic, idiomatic, cultural, and conceptual equivalence to the original version. The preliminary analysis revealed excellent intraobserver and interobserver reliability (ICC = 0.93; 95% CI: 0.83-0.97, p = 0.001 and ICC = 0.90; 95% CI: 0.74-0.96; p = 0.001, respectively). The items demonstrated adequate internal consistency, although ceiling and floor effects were considered beyond acceptable standards for some items. In the exploratory factor analysis, three factors were extracted that aggregated more than one construct to each component, but all were related to the “Activities and Participation” component of the International Classification of Functioning, Disability, and Health.

**Conclusion:**

The final version of the SATIS-Stroke scale in Brazilian Portuguese proved to be adequate and reliable for use on the Brazilian population. Further studies are underway to give continuity to the validation process and analyze the others measurement properties of the scale in the Brazilian population.

## 1. Introduction

According to the World Health Organization, stroke is the second major cause of death in the world and the third major cause of functional disability in worldwide [1]. With regard functioning, stroke survivors in Brazil demonstrate a moderate decline in participation and functional capacity [2]. After a stroke, the affected individual may have motor, sensory, perceptive, and cognitive impairments that exert an impact on functioning and lead to disability, with 24 to 75% of stroke survivors dependent on others for assistance with regard to activities of daily living [3–5]. These functional difficulties directly interfere with activities and social participation [6].

According to the International Classification of Functioning, Disability, and Health (ICF), participation is defined as involvement in real-life situations in society, whereas activity is defined as the capacity to perform the actions and tasks of daily living [7, 8]. Both constructs are considered important measures in the rehabilitation process, since activities and participation enable an individual to establish social relations, develop skills to meet social needs, and perform routine tasks and find purpose and meaning in life, which can have a positive impact on both physical and mental health [9, 10]. The decline in activity and social participation following a stroke has been demonstrated in the Brazilian population [2] and there is a need to monitor these constructs during the rehabilitation process.

Although the domains of the “Activities and Participation” component are presented in a single code list of the ICF, in clinical practice and for scientific purposes, these measures should be analyzed separately. For the classification and qualification of activities, it is necessary to use assessment tools that evaluate performance and functional capacity. However, such tests require adequate space to perform as well a previous training and a long application period [11, 12]. Among the measures available for the evaluation of social participation in the Brazilian population, such as the Assessment of Life Habits and the Stroke Specific Quality of Life scales, none addresses all domains of the ICF “Activities and Participation” component [13, 14]. Thus, there is a need for an assessment tool that is capable of measuring the activity and participation constructs in a single evaluation and that considers all ICF domains.

SATIS-Stroke was developed to measure satisfaction regarding activities and participation following a stroke and is considered to be a complete scale for this purpose [8, 12, 15]. However, this measure was originally published in French and, to date, has only been translated into English, with no version adapted to the Brazilian population. Therefore, the aim of the present study was to perform the translation and cross-cultural adaptation of SATIS-Stroke to Brazilian Portuguese, test its preliminary reliability and internal validity, and perform factor analysis using different samples of stroke survivors. The results obtained in this study are expected to contribute to the enhancement of the rehabilitation process for stroke survivors and facilitate the evaluation of functioning as recommended by the World Health Organization [9, 12].

## 2. Methods

### 2.1. Study Design and Ethical Aspects

A methodological, cross-sectional study was conducted involving the translation and cross-cultural adaptation of the SATIS-Stroke questionnaire to Brazilian Portuguese. This translation process received authorization from the authors who developed and validated the original questionnaire [15]. The study received approval from the Human Research Ethics Committee of University Nove de Julho, São Paulo, Brazil (certificate number: 74235417.7.0000.5511).

### 2.2. Sample Size

The sample size was determined following the recommendations proposed by Beaton et al. [16], who suggest that 40 individuals be evaluated in the pretest phase of a translated and adapted assessment measure. The analysis of preliminary reliability was based on the recommendations proposed by Hobart et al. [17], who determined a minimum of individuals for reliability studies involving patients with neurological disorders. For the factor analysis, we analyzed the answers of 80 individuals based on a Monte-Carlo simulation study by Barrett and Kline [18], who demonstrated that stable factorial solutions were found with a number ranging from 2 to 3 respondents per item. Thus, as the SATIS-Stroke had 36 items and considering a minimum of two respondents per item, the minimum n for factor analysis would be 72 participants.

### 2.3. Participants

All volunteers who met the inclusion criteria (clinical diagnosis of stroke at least six months earlier, age 20 years or older and hemiparesis) and agreed to participate in the present study signed a statement of informed consent. Individuals with hearing impairment, motor aphasia, comprehension aphasia, and cognitive impairment (based on the results of the Minimental State Examination) [19] were excluded from the study. All individuals were analyzed at the physical therapy outpatient clinics of University Nove de Julho.

### 2.4. Description of SATIS-Stroke

The SATIS-Stroke questionnaire is a fast, low-cost scale with 36 items distributed among the nine ICF domains and is applicable to all patients regardless of sex, age, marital status, affected side of the body, and phase of the poststroke period (acute, subacute, and chronic). The questionnaire is used to assess satisfaction, which regards the individual's perception of his/her performance in terms of meeting his/her needs during activities and situations of daily living. Such self-reported information assists in the planning of the rehabilitation process. Each item has four response options (very satisfied, satisfied, dissatisfied, and very dissatisfied). “Very dissatisfied” indicates that the individual is unable to perform the activity. “Dissatisfied” indicates that the individual is able to perform the activity, but not without assistance. “Satisfied” indicates that the individual is able to perform the activity alone, but with difficulty. “Very satisfied” indicates that the individual is able to perform the activity alone and with no difficulty. The items are scored from 0 to 3 points and the total ranges from 0 to 108 points, with higher scores denoting greater satisfaction with regard to activities and participation. Activities never performed or not performed in the previous month receive no score and are marked as "not applicable" [the respondent should select the question mark among the response options on the scale (see Supplementary Material)].

### 2.5. Procedures

#### 2.5.1. Translation and Cross-Cultural Adaptation

**Figure 1:**
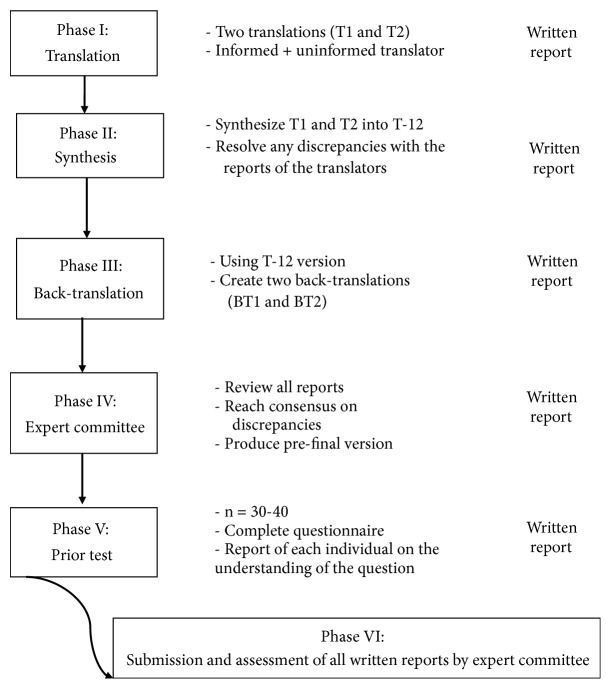
Steps of SATIS-Stroke translation and cross-cultural adaptation process.

The translation and cross-cultural adaptation of SATIS-Stroke were performed following internationally recommended guidelines [16] and consisted of six phases (Figure 1).

According to the recommendations proposed by Beaton et al. [16], the first step of the translation and cross-cultural adaptation process consists of the initial translation. In this phase, SATIS-Stroke was translated into Portuguese by two Brazilian translators fluent in both English and Portuguese. Translator 1 was informed with regard to the concepts on the questionnaire and Translator 2 was not informed. The two translators generated two independent versions of the questionnaire (T1 and T2).

In the second phase, the two translators evaluated the translations and synthesized a single version (T12). In the third phase, the synthesized version was back-translated into English by two translators fluent in both Portuguese and English, producing two independent back-translated versions (BT1 and BT2) for comparison to the original version to determine the validity of the translated version.

In the fourth step, the questionnaire was judged by a committee of experts in neurofunctional physical therapy, which was crucial to achieving cross-cultural equivalence. The role of the expert committee was to consolidate the translated versions and create the prefinal version of the questionnaire for the field tests. In the fifth step, a field test was conducted with the prefinal version using a sample of 40 individuals with a clinical diagnosis of stroke, as recommended by the guidelines for the cross-cultural adaptation of self-report measures. [16] The participants answered the translated version of the SATIS-Stroke questionnaire and were then interviewed to discuss their opinions regarding the limitations of the instrument, item by item, and any questions they may have about the scale.

In the sixth phase, all reports and the questionnaires filled out by the participants of the field test were presented to the committee along with the translated version to verify that all steps had been followed and that the reports adequately portrayed the process.

#### 2.5.2. Reliability

The preliminary reliability of SATIS-Stroke was evaluated on two occasions. For the determination of intraobserver agreement, test-retest analysis was performed with an interval of seven to 14 days between evaluations. For the determination of interobserver agreement, a second observer applied the questionnaire after a period of seven to 14 days. The minimum interval of seven days was necessary to avoid the memorization of the answers and the maximum interval of 14 days was used to minimize the possibility of a change in the respondent's satisfaction regarding activities and participation [20].

### 2.6. Statistical Analysis

The Shapiro-Wilk test was used to determine the normality of the data. Parametric data were expressed as mean and standard deviation. Nonparametric data were expressed as median and interquartile range. Categorical variables were described in absolute values and percentage of the total sample. The intraclass correlation coefficient (ICC) and respective 95% confidence intervals were used for the analysis of reliability. Intraobserver and interobserver reliability were tested using the following classification: ICC < 0.40 = weak reliability; 41 to 0.75 = moderate reliability; and > 0.75 = excellent reliability [21, 22].

Ceiling and floor effects were calculated based on the percentage of volunteers who had the highest (ceiling) and lowest (floor) scores. These effects are considered when at least 15% of the individuals have the maximum or minimum scores. [20]

Cronbach's alpha (*α*) was used for the analysis of the internal consistency of the SATIS-Stroke. The coefficients were interpreted as follows 0.90 to 0.95 = very good; 0.80 to < 0.90 = good, 0.70 to <0.80 = fair, 0.60 to < 0.70 = weak, and < 0.60 = unacceptable. [23]

Two evaluation methods were employed to analyze whether the data matrix could be submitted to factorization: the Kaiser-Meyer-Olkin (KMO) criterion and Bartlett's Sphericity Test. As a rule for the interpretation of the KMO indices, values lower than 0.5 are considered unacceptable, values between 0.5 and 0.7 are considered mediocre, values between 0.7 and 0.8 are considered good, and values higher than 0.8 and 0.9 are considered very good and excellent, respectively [24]. Principal component analysis with varimax orthogonal rotation was used for the extraction of the data. As the aim of factor analysis is to reduce the number of variables into a smaller number of factors, only factors with an* eigenvalue* > 2 were retained for analysis [25]. Only items with a factorial load ≥ 0.35 (positive or negative) were included in the factors.

The data were analyzed using the SPSS program, version 22, and a 5% level of significance was adopted for all analyses (p < 0.05).

## 3. Results

### 3.1. Characteristics of Participants

Forty-eight stroke survivors were recruited, eight of whom were excluded for aphasia. Therefore, the sample was composed of 40 individuals.  Table 1 displays the demographic and clinical characteristics of the participants.

### 3.2. Translation and Cross-Sectional Adaptation

The translation process from phases I to III occurred with the collaboration of four translators (T1, T2, BT1, and BT2). No disagreements were found among the two translators who performed the versions in Portuguese or between the two translators who performed the back-translations into English. All versions were sent to the expert committee. In phase IV, the expert committee decided to change verbs in the gerund to the infinitive for all items on the questionnaire to satisfy the grammar rules of the Portuguese language. Some words were changed to achieve semantic and conceptual equivalence. For example, on Item 1 “participating in the preparation of foods and beverages in all circumstances”, the word “circumstances” was changed to “situations” (Table 2).

The committee also decided to add or replace some words with synonyms on 13 items of the questionnaire to facilitate its understanding by the Brazilian population. The items that required the use of synonyms are listed in Table 3 with the original version in English, the synthesized translated version, and the definitive version established by the expert committee.

In phase V, the prefinal version of the SATIS-Stroke questionnaire was administered to 40 individuals with hemiparesis stemming from a stroke. In phase VI, these individuals were asked if they understood all the questions and demonstrated no difficulties understanding the questionnaire in the form that was administered as the prefinal version defined by the expert committee. The final version of the questionnaire is attached (Supplementary Material 1).

### 3.3. Preliminary Reliability

Table 4 displays the ICC values and mean differences regarding intraobserver and interobserver reliability. Excellent agreement was found in both cases and all results were significant (p = 0.001).

### 3.4. Internal Consistency and Ceiling and Floor Effect

Items 8, 17, 35, and 36 were those that most addresses activities that were either never performed or not performed in the previous 30 days and were therefore marked as not applicable (N/A). The ceiling and floor effects were higher than acceptable standards (greater than 15%) for some items. Adequate internal consistency was found for the score of the SATIS-Stroke items (Table 5).

### 3.5. Exploratory Factor Analysis

Preliminary analyses of the 36 items on the scale indicated missing data due to the use of the N/A option (not applicable = activity never performed or not performed in previous 30 days). For these missing data, the mean of the respective item was used. Frequency and distribution analyses of the 36 items indicated satisfactory indices with regard to dispersion and kurtosis as well as the absence of univariate discrepant cases.

The extraction of the principal components revealed the goodness-of-fit of the correlation matrix regarding the necessary presuppositions for multivariate analysis, such as the absence of multicollinearity and the factorability of the data. The KMO indicator was 0.770. Bartlett's Sphericity Test indicated p=0.001. Therefore, factorization of the data was possible. Values > 0.50 were found in the analyses of commonalities, indicating that the percentage of variance of each variable explained by common factors was higher than 50% in all cases.

Considering* eigenvalues *> 2 and the scree plot, the decision was made to extract three factors using principal component analysis with varimax orthogonal rotation. Only items with a load ≥ 0.35 (positive or negative) were included in the factors. The extracted components in the analysis explained 45.50% of the total variability in the data. The first factor explained 25.05%, the second factor explained 13.94%, and the third factor explained 6.50%. The saturations of each item in the respective factors are displayed in Table 6 and are arranged based on the factor load rather than their order on the scale. For cases in which an item could pertain to more than one factor, the criterion of the highest saturation values was used first, followed by the semantic content of the item and its pertinence to the previously described factor.

## 4. Discussion

The purpose of the present study was to perform the translation and cross-sectional adaptation of SATIS-Stroke to Brazilian Portuguese, test its preliminary reliability and internal validity, and perform factor analysis of the measure using different samples of stroke survivors. The analysis of the results revealed that the final translated version of the scale is adequate and reliable for use on the Brazilian population.

The sample analyzed was predominantly composed of men with a mean age of 57 years and more than 60 months since the occurrence of the stroke event. Among these characteristics, time elapsed since a stroke is the variable that is most related to poststroke participation [2]. No significant differences were found regarding the affected side of the body, but higher frequencies were found of ischemic stroke and married individuals. Mean schooling was 2.5 years. These findings are in agreement with data in previous studies reporting that the population of stroke survivors in Brazil has a low level of schooling [2].

During phases I, II, and III, no disagreements occurred during the translation from English into Portuguese. In phase IV (meeting of the expert committee), the main changes in the translation process were performed to achieve semantic and conceptual equivalence on the questionnaire: the word “circumstances” was changed to “situations”. The aim of these changes was to facilitate the understanding of the target population, considering the sociocultural characteristics of Brazilian stroke survivors, who have a low level of schooling [2], as confirmed in the sample analyzed in the present study.

In phase IV, 13 of the 36 items on the questionnaire were altered with synonyms to facilitate the understanding on the part of the Brazilian population and to be in accordance with the idiomatic properties of Brazilian Portuguese. When divergences of opinion occurred regarding which synonyms to use, the concepts of the “Activities and Participation” component of the ICF were used as reference, since the questionnaire was designed based on this classification. All changes are displayed in Table 3 and occurred to facilitate the interpretation of the questionnaire by Brazilian stroke survivors. Item 27, which was first translated as “using storage space in your house”, was changed to “use spaces in your home to store food, beverages, closed and other objects necessary to daily living”. The aim was to improve the understanding of the item, as the original statement was very broad, which could lead to interpretation errors. Therefore, the decision was made to discriminate what objects could be stored. The choice of these objects was based the description of category d6404 of the “Activities and Participation” component of the ICF.

In phase VI, the patients reported no difficulties understanding the items on the translated version of the questionnaire. Upon receiving the explanations regarding the response options at the onset of the evaluation, the participants demonstrated a good understanding and had no difficulties answering the questionnaire.

According to Portney and Watkins [26], reliability is the first prerequisite of a measure and regards its consistency. Reliability studies are crucial to determining the variability of a method or assessment tool as well as avoiding interpretation errors regarding the variables before and after an intervention. The translated version of the SATIS-Stroke exhibited excellent reliability, as demonstrated by the high levels of intraobserver and interobserver agreement. These findings are similar to those described by Bouffiloux et al. [15], who report excellent test-retest reliability when analyzing the original version of the questionnaire.

On Item 8, the evaluators noticed that most of the individuals responded that they did not participate in events involving arts and culture. This was likely related to the cultural and socioeconomic level of the volunteers, who were recruited from low-income communities and therefore had restrictions with regard to attending cultural activities and events.

The same was found for items 17 “Participating in spousal relationships”; 35 “Maintaining emotional relationships”; and 36 “Having a sexual relationship with somebody”, demonstrating that the sample analyzed has problems with emotional, intimate, and sexual relationships, even though the majority of participants were married. Similar results are described by Silva et al. [27], who analyzed the response theory to the items on the SS-QOL for the evaluation of participation and identified that the most erratic item on the scale was that which addressed the issue of sexual relations. The literature indicates that sexual dysfunction and diminished sexual satisfaction are common among stroke survivors and are associated with physical, psychosocial, and relationship factors [28]. However, these aspects are not adequately addressed in rehabilitation programs for this population. Therefore, these items should not be removed from the SATIS-Stroke questionnaire, since they address important issues regarding satisfaction with interpersonal relationships.

The total SATIS-Stroke score did not exhibit ceiling or floor effects, but such effects were beyond acceptable standards for some items. In general, more items had a ceiling effect than a floor effect. It is likely that the chronic nature of a stroke led to greater acceptance and adaptation on the part of the participants, leading to higher satisfaction scores. Silva et al. [9] report similar findings. Despite these ceiling and floor effects for some items, all items on the SATIS-Stroke demonstrated excellent internal consistency (Cronbach's *α*: 0.90 to 0.95), which lends further support to the internal reliability of the SATIS-Stroke.

In general, the 36 items on the SATIS-Stroke questionnaire are related to the nine domains of the "Activities and Participation" of the ICF. However, principal component analysis led to the extraction of three factors that aggregated more of one construct to each of the factors identified. Considering the semantic content of the items and the relationship between the SATIS-Stroke questionnaire and the ICF, the first factor regards activities of personal care, mobility, home life, and the main aspects of life; the second factor addresses issues regarding community, social and civic life, learning, and the application of knowledge as well as general tasks and demands; and the third factor regards communication, interactions, and interpersonal relationships.

Despite the important information presented, this study has limitations that should be pointed out. As a preliminary study, the sample size was not large and the results should therefore be interpreted with caution. Further studies are needed for a more in-depth examination of the validity and reproducibility of the SATIS-Stroke questionnaire using a larger sample. Despite these limitations, the study provides the translated and adapted version of an important measure used in both clinical practice and scientific research, thereby contributing to a better evaluation and therapeutic follow up of stroke survivors.

## 5. Conclusion

The final Brazilian version of the SATIS-Stroke questionnaire exhibited adequate semantic, idiomatic, cultural, and conceptual equivalence to the original version as well as adequate preliminary reliability. These findings demonstrate that SATIS-Stroke is a promising assessment tool for the evaluation of satisfaction among stroke survivors with regard to activities and social participation. However, studies addressing the validation and other properties of this questionnaire are currently underway to enable a more accurate analysis.

## Figures and Tables

**Table 1:** Demographic and clinical characteristics of participants.

Variable	(n = 40)
*Sex*	
Male	25 (62.5%)
Female	15 (37.5%)
*Age*	57.50 ± 11.86
*Time since stroke (months)*	60.13 ± 69.63
*Affected side of body*	
Right	20 (50%)
Left	20 (50%)
*Type of stroke*	
Ischemic	32 (80%)
Hemorrhagic	8 (20%)
*Marital status*	
Married	19 (47.5%)
Single	10 (25%)
Widowed	6 (15%)
Divorced	5 (12.5%)
*Schooling (years)*	2.63 ± 8.3
*Mini Mental State Examination*	25.15 ± 3.42

Data expressed as absolute and relative frequency; mean and standard deviation for parametric data; median and interquartile range (25% and 75%) for nonparametric variables.

**Table 2:** Items altered to achieve semantic and conceptual equivalence.

Synthesis of translated versions	Definitive version
*1- Participando no preparo de alimentos e bebidas em todas as circunstâncias. *	*1- Participar no preparo de alimentos e bebidas em todas as * ***situações***.
*2- Usando faca, garfo e colher em todas as circunstâncias*	*2- Usar faca, garfo e colher em todas as * ***situações***.
*13- Gerenciando seus rendimentos em todas as circunstâncias. *	*13- Administrar seus rendimentos em todas as * ***situações***.
*14- Usando moedas e notas de dinheiro em todas as circunstâncias.*	*14- Usar moedas e notas de dinheiro em todas as * ***situações***.
*21- Preencher informações em documentos/formulários em todas as circunstâncias.*	*21-Preencher informações em documentos/formulários em todas as * ***situações***.
*23- Movendo-se para fora de sua casa em todas as circunstâncias. *	*23- Mover-se para fora de casa em todas as * ***situações***.
*34- Gerenciando suas dores em todas as circunstâncias.*	*34- Controlar suas dores em todas as * ***situações.***

*∗* Changed items are in bold.

**Table 3:** Items altered during translation and cross-cultural adaptation process.

Original version	Synthesis of translated versions	Version defined by expert committee
3- Participating in spoken exchange of information with your entourage.	*3- Participando na troca de informações verbais com o ambiente.*	*3- Participar de * ***conversas com seus amigos.***
5- Undressing to use the toilet and redressing in your home or outside of this one.	*5- Despindo-se para usar o banheiro e vestindo-se em sua casa e fora deste.*	*5- * ***Despir-se e vestir-se*** * para usar o vaso sanitário em sua casa e fora dela.*
7- Having an urinary continence in your home and outside of this one.	*7- Ter uma continência urinária em casa e fora deste.*	*7- * ***Em controlar sua urina*** * em sua casa e fora dela.*
8- Participating in arts and culture (cinema, theatre, etc.)	*8- Participando em artes e cultura (cinema, teatro, etc.)*	*8- Participar de * ***eventos e locais*** * de artes e cultura.*
9- Co-operating with your entourage.	*9-Cooperando com outras pessoas.*	*9- * ***Ajudar*** * outras pessoas.*
13- Managing your incomes in all circumstance.	*13- Gerenciando seus rendimentos em todas as circunstâncias.*	*13- * ***Administrar*** * seus rendimento em todas as situações.*
17- Participating in spousal relationships.	*17- Participando de relações conjugais.*	*17- Participar de relações * ***românticas e intimas com seu parceiro (a).***
19- Reaching objects in your closely space.	*19- Alcançando objetos em seu espaço próximo.*	*19- Alcançar objetos * ***ao seu redor.***
21- To supplement administrative documents in all circumstance.	*21- Ler e compreender um documento em todas as circunstâncias.*	*21- * ***Preencher informações*** * em documentos/ * ***formulários*** * em todas as situações.*
27- Using storage spaces in your house.	*27- Usando espaços de armazenamento em sua casa.*	*27- Usar os espaços de sua casa para * ***armazenar alimentos, bebidas, roupas e outros objetos necessários para seu dia-a-dia.***
28- Choosing appropriate clothes.	*28- Escolhendo roupas apropriadas.*	*28- Escolher roupas apropriadas * ***de acordo com a ocasião.***
34- Managing your pains in all circumstance.	*34- Gerenciando suas dores em todas as circunstâncias.*	*34- * ***Controlar*** * suas dores em todas as situações.*
36- Having a sexual relationship with another.	*36- Tendo um relacionamento sexual com outro.*	*36- Ter um relacionamento sexual com * ***seu parceiro (a).***

*∗* The words added in each item are in bold.

**Table 4:** Preliminary reliability of Brazilian version of SATIS-Stroke (intra-observer and inter-observer agreement) (n = 40).

ICC_2,1_	Reliability (95% CI)	MD ± SD
Intra-observer	0.91 (0.83-0.95)	-1.85± 9.57
Inter-observer	0.91 (0.82-0.93)	-1.87± 9.83

ICC: intraclass correlation coefficient; CI: confidence interval; MD: mean difference; SD: standard deviation; *∗*p = 0.001 for all ICCs.

**Table 5:** Internal consistency and both ceiling and floor effects for SATIS-Stroke items.

SATIS-Stroke items	N/A option obtained (%)	Ceiling effect (%)	Floor effect (%)	Cronbach's *α*
1. Participating in food and drink preparation in all circumstances.	8.75	10	*17.5*	0.93
2. Using knife, fork and spoon in all circumstances.	-	11.3	7.5	0.94
3. Participating in spoken exchange of information with your entourage.	-	*40*	5	0.94
4. Washing your hair according to your needs.	-	*21.3*	6.3	0.94
5. Undressing to use the toilet and redressing in your home or outside of it.	-	15	8.8	0.94
6. Carrying out your personal hygiene according to your needs.	-	*21.3*	7.5	0.94
7. Having urinary continence in your home and outside of it.	-	*28.8*	7.5	0.94
8 Participating in arts and culture (cinema, theatre, etc.).	*53.75*	13.8	5	0.94
9. Co-operating with your entourage.	1.25	*50*	7.5	0.94
10. Reading and understanding a document in all circumstances.	-	*17.5*	6.3	0.94
11. Using the telephone at home according to your needs.	-	*21.3*	5	0.94
12. Listening to and watching television according to your needs.	-	*58.8*	6.3	0.94
13. Managing your income in all circumstances.	1.25	*21.3*	5	0.94
14. Using coins and banknotes in all circumstances.	1.25	*20*	6.3	0.94
15. Dressing and undressing in all circumstances and according to your needs.	-	13.8	10	0.94
16. Ensuring that your rights are respected.	-	*31.8*	5	0.94
17. Participating in spousal relationships.	*22.5*	12.5	*20*	0.93
18. Taking a bath or your shower according to your needs.	-	*22.5*	5	0.93
19. Reaching objects in your near space.	-	12.5	2.5	0.93
20. Getting clothes out of the closet.	1.25	13.8	15	0.93
21. To supplement administrative documents in all circumstances.	-	5	*27.5*	0.94
22. Moving inside your home.	-	*21.3*	7.5	0.93
23. Moving outside your home in any circumstances.	-	8.8	*22.5*	0.94
24. Climbing and going downstairs all stages in your home according to your needs.	-	6.3	13.8	0.94
25. Entering and exiting your home according to your needs.	-	13.8	6.3	0.93
26. Opening and closing doors in your home.	-	*17.5*	3.8	0.93
27. Using storage spaces in your house.	3.75	11.3	12.5	0.93
28. Choosing appropriate clothes.	-	*22.5*	1.3	0.94
29. Getting feelings across.	-	*16.3*	3.8	0.94
30. Being aware of what surrounds you.	-	*38.8*	1.3	0.94
31. Expressing oneself to someone.	-	*18.8*	1.3	0.94
32. Participating in ceremonies (marriage, gathering family, etc.).	8.75	*20*	3.8	0.94
33. Asking for help in an emergency situation.	-	11.3	2.5	0.94
34. Managing your pain in all circumstances.	1.25	10	8.8	0.94
35. Maintaining emotional relationships.	*15*	8.8	11.3	0.94
36. Having a sexual relationship with somebody.	*28.75*	11.3	*21.3*	0.94

N/A: not applicable = activity never performed or not performed in previous 30 days.

**Table 6:** Rotated Component Matrix (n=80).

	Factor		
SATIS-Stroke Items	1	2	3
1. Participating in food and drink preparation in all circumstances.	.576		
2. Using knife, fork and spoon in all circumstances.	.429		
4. Washing your hair according to your needs.	.615		
1. Undressing to use the toilet and redressing in your home or outside of it.	.771		
6. Carrying out your personal hygiene according to your needs.	.679		
7. Having urinary continence in your home and outside of it.	.491		
9. Co-operating with your entourage.	.451		
13. Managing your income in all circumstances.	.510		
14. Using coins and banknotes in all circumstances.	.599		
15. Dressing and undressing in all circumstances and according to your needs.	.746		
18. Taking a bath or your shower according to your needs.	.744		
19. Reaching objects in your near space.	.385		
20. Getting clothes out of the closet.	.646		
22. Moving inside your home.	.821		
23. Moving outside your home in any circumstances.	.639		
24. Climbing and going downstairs all stages in your home according to your needs.	.692		
25. Entering and exiting your home according to your needs.	.807		
26. Opening and closing doors in your home.	761		
27. Using storage spaces in your house.	.587		
28. Choosing appropriate clothes.	.460		
34. Managing your pain in all circumstances.	.458		
8 Participating in arts and culture (cinema, theatre, etc.).		.386	
10. Reading and understanding a document in all circumstances.		.679	
12. Listening to and watching television according to your needs.		.643	
16. Ensuring that your rights are respected.		.414	
21. To supplement administrative documents in all circumstances.		.539	
32. Participating in ceremonies (marriage, gathering family, etc.).		.584	
33. Asking for help in an emergency situation.		.578	
3. Participating in spoken exchange of information with your entourage.			.499
11. Using the telephone at home according to your needs.			.456
17. Participating in spousal relationships.			.354
29. Getting feelings across.			.554
30. Being aware of what surrounds you.			.480
31. Expressing oneself to someone.			.735
35. Maintaining emotional relationships.			-.408
36. Having a sexual relationship with somebody.			-.506

Extraction Method: Principal Component Analysis.

Rotation Method: Varimax with Kaiser Normalization.

## Data Availability

The data used to support the findings of this study are included within the article and in the supplementary information file.

## Supplementary Materials

Supplementary Materials Final Brazilian Portuguese version of SATIS-Stroke.
