# Supplementary material for: Translation and Cross-Cultural Adaptation of SATIS-Stroke for Use in Brazil: A Satisfaction Measure of Activities and Participation in Stroke Survivors
Source: Biomed Res Int. 2019 Feb 18;2019:8054640. doi: 10.1155/2019/8054640 (PMC6398040; doi:10.1155/2019/8054640)
Supplement: Supplementary Materials — Final Brazilian Portuguese version of SATIS-Stroke. [file 8054640.f1.pdf]

SUPPLEMENTARY MATERIAL

Final Brazilian Portuguese version of SATIS-Stroke.

SATIS-Stroke: Satisfação de Atividade e Participação para Acidente Vascular Encefálico (Versão em português)

Paciente:____**Data:**______________

	Qual é o seu NÍVEL DE SATISFAÇÃO na realização das seguintes situações de vida?	Muito Insatisfeito	Insatisfeito	Muito Satisfeito	Satisfeito	?
1	Participar no preparo de alimentos e bebidas em todas as situações.					
2	Usar faca, garfo e colher em todas as situações.					
3	Participar de conversas com seus amigos.					
4	Lavar seus cabelos de acordo com as suas necessidades.					
5	Despir-se ou vestir-se para usar o vaso sanitário em sua casa ou fora dela.					
6	Fazer a sua higiene pessoal de acordo com as suas necessidades.					
7	Em controlar sua urina em sua casa e fora dela.					
8	Participar de eventos e locais de artes e culturas.					
9	Ajudar outras pessoas.					

10	Ler e compreender um documento em todas as situações.					
11	Usar o telefone em casa de acordo com suas necessidades.					
12	Ouvir e assistir à televisão de acordo com suas necessidades.					
13	Administrar seus rendimentos em todas as situações.					
14	Usar moedas e notas de dinheiro em todas as situações.					
15	Vestir-se e despir-se em todas as situações e de acordo com suas necessidades.					
16	Garantir que seus direitos sejam respeitados.					
17	Participar de relações românticas e íntimas com seu parceiro(a).					
18	Tomar seu banho em seu chuveiro de acordo com suas necessidades.					
19	Alcançar objetos ao seu redor.					
20	Tirar roupas do armário.					
21	Preencher informações em documentos/formulários em todas as situações.					
22	Mover-se dentro de sua casa.					
23	Mover-se para fora de sua casa em todas as situações.					
24	Subir e descer todos os andares em sua casa de acordo com suas necessidades.					
25	Entrar e sair de sua casa de acordo com suas necessidades.					

26	Abrir e fechar as portas em sua casa.					
27	Usar os espaços de sua casa para armazenar alimentos, bebidas, roupas e outros objetos necessários para o seu dia-a-dia.					
28	Escolher roupas apropriadas de acordo com a ocasião.					
29	Compartilhar seus sentimentos.					
30	Estar consciente com o que está ao seu redor.					
31	Expressar-se para alguém.					
32	Participar de cerimônias (casamento, reunião de família, etc.).					
33	Pedir ajuda em uma situação de emergência.					
34	Controlar suas dores em todas as situações.					
35	Manter-se em relacionamentos afetivos.					
36	Ter um relacionamento sexual com seu parceiro(a).					

Instruções para o questionário SATIS-Stroke

<u>O questionário SATIS-Stroke</u>

O questionário SATIS-Stroke foi desenvolvido como uma medida de satisfação da atividade e participação percebida pelo paciente. Com o intuito de encontrar a satisfação das atividades e situações de vida mais específica. Alguns itens foram selecionados a partir de escalas. A primeira aplicação do questionário em uma amostra de pacientes com AVC mostrou que os itens definidos apontam uma escala de satisfação válida. O SATIS-Stroke foi desenvolvido originalmente usando o modelo de medição Rasch. Isto permite converter pontuações ordinais em medidas lineares localizadas em uma escala unidimensional.

Procedimentos

O questionário SATIS-Stroke Brasil deve ser administrado sob forma de entrevista. Os pacientes são solicitados a estimar o nível de satisfação na realização de cada atividade e situação de vida, quando são feitas:

- No mês do preenchimento do questionário;

- Com outra ajuda técnica ou humana (mesmo que o paciente realmente use ajuda na vida diária);

- Qualquer que seja a estratégia usada (qualquer compensação é permitida).

Durante a avaliação, uma escala de resposta de 4 níveis é apresentada aos pacientes. Os pacientes são convidados a avaliar sua percepção na escala de respostas como "Muito insatisfeito", "Insatisfeito", "Satisfeito" ou "Muito satisfeito". Atividades não realizadas no último mês não são pontuadas e são inseridas como Não Aplicável (marque o ponto de interrogação). Para qualquer atividade as quatro respostas possíveis são:

- Muito insatisfeito: o paciente é incapaz de realizar a atividade sem usar qualquer outra ajuda;

- Insatisfeito: o paciente é capaz de realizar sozinho, mas com um pouco de ajuda;

- Satisfeito: o paciente consegue realizar a atividade sozinho, mas apresenta dificuldades

- Muito satisfeito: o paciente realiza a atividade sozinho, sem nenhuma dificuldade.

- Não Aplicável ou Ponto de interrogação: o paciente não pode estimar a satisfação da atividade porque ele nunca fez a atividade ou não a realizou no período de um mês.

Note que quando um paciente nunca tentou a atividade, o avaliador precisa ter certeza se o indivíduo nunca realizou. As instruções são dadas ao paciente apenas no início do teste. Cinco itens são usados para treinamento, a fim de ajudar o paciente a sentir cada nível da escala de classificação e usar toda a amplitude da escala de resposta. O examinador pode repetir as instruções sempre que o paciente mostrar alguma hesitação em responder.
